# Author Correction: DECIMER.ai: an open platform for automated optical chemical structure identification, segmentation and recognition in scientific publications

**DOI:** 10.1038/s41467-023-41814-5

**Published:** 2023-09-27

**Authors:** Kohulan Rajan, Henning Otto Brinkhaus, M. Isabel Agea, Achim Zielesny, Christoph Steinbeck

**Affiliations:** 1 Institute for Inorganic and Analytical Chemistry, Friedrich Schiller University Jena, Lessingstr. 8, 07743 Jena, Germany; 2 Department of Informatics and Chemistry, Faculty of Chemical Technology, University of Chemistry and Technology Prague, Technicka 5, 166 28 Prague, Czech Republic; 3 Institute for Bioinformatics and Chemoinformatics, Westphalian University of Applied Sciences, August-Schmidt-Ring 10, 45665 Recklinghausen, Germany

**Keywords:** Cheminformatics, Organic chemistry, Data mining, Literature mining, Machine learning

Correction to: *Nature Communications* 10.1038/s41467-023-40782-0, published online 19 August 2023

The original version of this Article contained an error in Fig. 3, panels A and B, in which the legend 'Mean- Proportion of Identical Prediction' was erroneously associated to the blue symbols and shading, instead of orange, and the legend 'Mean—Tanimoto Similarity' was associated to the orange symbols and shading, instead of blue. This has been corrected in both the PDF and HTML versions of the Article.

