# Supplementary material for: DECIMER.ai: an open platform for automated optical chemical structure identification, segmentation and recognition in scientific publications
Source: Nat Commun. 2023 Aug 19;14:5045. doi: 10.1038/s41467-023-40782-0 (PMC10439916; doi:10.1038/s41467-023-40782-0)
Supplement: Supplementary file 1 — Supplementary Information [file 41467_2023_40782_MOESM1_ESM.pdf]

Supplementary Information

List of Abbreviations

AUC - Area Under the Curve
BLEU - BilinguaL Evaluation Understudy
CDK - Chemistry Development Kit
ChEBI - Chemical Entities of Biological Interest
CLEF - Conference and labs of the evaluation forums
CLIDE - Chemical literature data extraction
CNN - Convolutional Neural Networks
COCONUT - COlleCtion of Open Natural prodUcTs
CXSMILES - ChemAxon Extended SMILES
DECIMER - Deep lEarning for Chemical IMagE Recognition
FLOPS - Floating Point Operations Per Second
FN - False Negative
FP - False Positive
FTP - File Transfer Protocol
GCP - Google Cloud Platform
GPU - Graphical Processing Unit
InChI - International Chemical Identifier
J - Youden index
JNP - Journal of Natural Products
JPO - Japanese Patent Office
MCC - Matthews Correlation Coefficient
NP - Natural Products
OCSR - Optical Chemical Structure Recognition
OSRA - Optical Structure Recognition Application
PIKAChU - Python-based Informatics Kit for Analysing CHemical Units
PDF - Portable Document Format
PNG - Portable Network Graphics
PyPI - Python Package Index
R-CNN - Region-Based Convolutional Neural Networks
R-group - Rest group
ROC - Receiver Operating Characteristic
SE - Squeeze and Exit
SELFIES - Self-referencing embedded strings
SMILES - Simplified Molecular-Input Line-Entry System
STOUT - SMILES-TO-IUPAC-name Translator
TFRecord - TensorFlow Record file
TN - True Negative
TP - True Positive
TPU - Tensor Processing Unit
UOB - University of Birmingham, United Kingdom (dataset)
USPTO - United States Patent and Trademark Office

VM - Virtual Machine

Training and test of the *DECIMER Image Transformer* 299 x 299 model

For the models trained using datasets containing images with a size of 299 x 299 pixels, EfficientNet-V2-B3 was used without any further modifications. Training the model with the image size of 299 x 299 pixels was done using the TPU v3-32 pod slices. The per-node batch size was set to 128 for the models trained on datasets with images of 299 x 299 pixels. Training scripts and models are written in Python 3 with Keras and Tensorflow 2.8.0.

To test each model trained with the *pubchem_1* and *pubchem_2* datasets with an image size of 299 x 299 pixels, a pre-selected set of 250,000 molecules was used. Each of these molecules was depicted twice with an image size of 299 x 299 pixels, once without augmentations and once with augmentations. The model trained using *pubchem_2* was further evaluated by a set of 250,000 Markush structures, depicted with and without augmentations. The purpose of this was to evaluate the model's accuracy in detecting chemical structures depicted with R-Group representations.

Supplementary Table 1. Training datasets for *DECIMER Image Transformer*

Dataset ID	Number of molecules	Number of depictions	Composition
pubchem_1	107.5 Mio	430 Mio	1 clean and 3 augmented depictions per molecule with a size of 299 x 299 pixels
pubchem_2	127.5 Mio	479.5 Mio	All molecules from *pubchem_1* + 20 Mio depictions of Markush structures with a resolution of 299 x 299 pixels<br>(1 clean and 3 augmented depictions per molecule)
pubchem_3	127.5 Mio	453.9 Mio	All molecules and Markush structures from *pubchem_2* with a resolution of 512 x 512 pixels<br>(1 clean and 3 augmented depictions per molecule)
hand_drawn	127.5 Mio	127.5 Mio	1 depiction with augmentations with a resolution of 512 x 512 pixels that makes it appear like hand-drawn per molecule.

Supplementary Table 2. Test results analysis and model performance. Columns A, B and C contain the test results on images with a resolution of 299 x 299 pixels. Columns D and E contain the test results on images with a resolution of 512 x 512 pixels. Finally, columns F and G contain the BLEU scores for the test results from C and E.

	A		B		C	
Test data	299 x 299 depictions (no Markush structures)		299 x 299 depictions (no Markush structures)		299 x 299 depictions (with Markush structures)	
Model trained on	*pubchem_1* (no Markush structures)		*pubchem_2* (with Markush structures)		*pubchem_2* (with Markush structures)	
	Non-Augmented Images	Augmented Images	Non-Augmented Images	Augmented Images	Non-Augmented Images	Augmented Images
Valid Predictions	99.79%	99.56%	96.50%	96.60%	99.80%	99.79%
Identical Predictions	90.85%	85.62%	86.68%	80.58%	73.23%	54.07%
Tanimoto 1.0 Count	95.66%	90.68%	92.89%	88.21%	91.89%	83.07%
Average Tanimoto	0.99	0.98	0.96	0.95	0.99	0.97

	D		E	
Test data	512 x 512 depictions (without Markush structures)		512 x 512 depictions (with Markush structures)	
Model trained on	*pubchem_3*		*pubchem_3*	
	Non-Augmented Images	Augmented Images	Non-Augmented Images	Augmented Images
Valid Predictions	96.46%	96.54%	99.83%	99.81%
Identical Predictions	91.24%	89.65%	81.06%	74.65%
Tanimoto 1.0 Count	94.77%	93.47%	94.42%	92.06%
Average Tanimoto	0.96	0.96	0.99	0.99

BLEU Scores for test results with R-Group representations.

	F		G	
Test data	299 x 299 depictions (with Markush structures)		512 x 512 depictions (with Markush structures)	
Model trained on	*pubchem_2*		*pubchem_3*	
BLEU Scores	Non-Augmented Images	Augmented Images	Non-Augmented Images	Augmented Images
BLEU-1:	0.96	0.95	0.97	0.97
BLEU-2:	0.96	0.94	0.97	0.96
BLEU-3:	0.95	0.93	0.96	0.96
BLEU-4:	0.94	0.91	0.96	0.95
Average	0.94	0.91	0.96	0.95

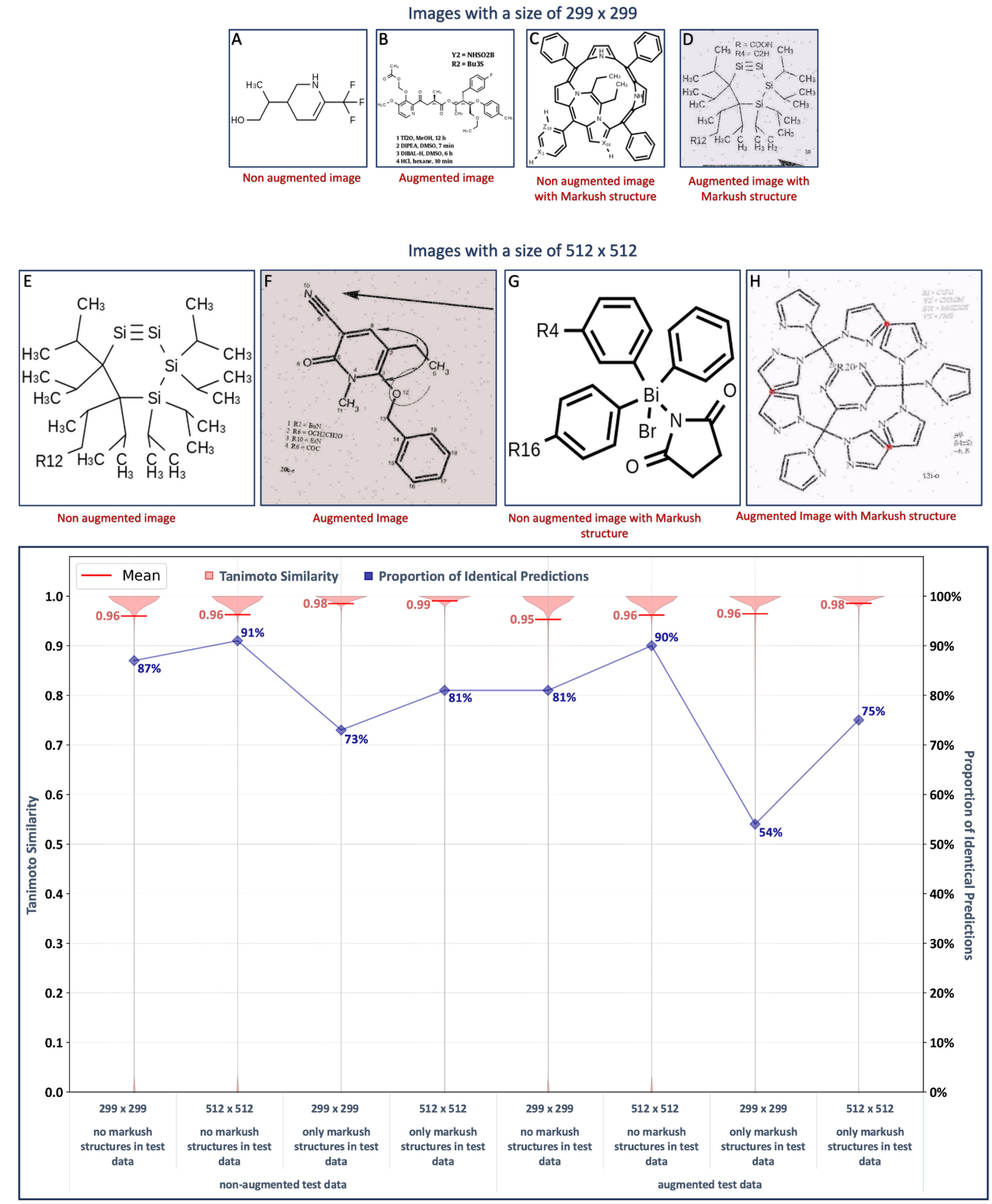

Supplementary Figure 1: Representation of types of images in the training and the test datasets and test results. Images with a size of 299 X 299 - A: Non-augmented Image, B: Augmented image, C: Non-augmented image with Markush structure and D: Augmented image with

Markush structure. Images with a size of 512 X 512 - E: Non-augmented Image, F: Augmented image, G: Non-augmented image with Markush structure and H: Augmented image with Markush structure. I: In-domain test results of two models trained and tested using images with a resolution of 299 x 299 or 512 x 512 pixels, respectively. All training datasets include depictions of Markush structures and a variety of image augmentations (datasets pubchem_2 and pubchem_3 in Supplementary Table 1). In the test datasets, these features were separately evaluated as described in the text to assess their influence on performance. All in-domain test results are also presented in Supplementary Table 2.

Supplementary Table 3: Evaluation of the effect of the representation of R-group indices in the training data. Test performance of a model trained on SMILES strings without further modifications (Model 1) and SMILES strings with replaced R-group indices (Model 2)

	Model 1 (SMILES)	Model 2 (modified SMILES)
Valid Predictions	95.70%	99.33%
Identical Predictions	77.87%	77.37%
Tanimoto 1.0 Count	86.55%	88.78%
Average Tanimoto similarity	0.94	0.98

Supplementary Table 4. Datasets used as non-chemical structures to train, validate and test the DECIMER Image Classifier.

Dataset name	Number of images	Modifications	Reference
Places-205	2462123	None	B. Zhou, A. Lapedriza, J. Xiao, A. Torralba, and A. Oliva. Learning Deep Features for Scene Recognition using Places Database. Advances in Neural Information Processing Systems 27 (NIPS), 2014.
COCO	287360	None	Lin, Tsung-Yi et al. (2014). Microsoft

			COCO: Common Objects in Context. https://arxiv.org/abs/1405.0312
Google Open labelled Images	1909039	None	https://storage.googleapis.com/openimages/web/index.html
MMU-OCR-21	301229	None	T. Nasir, M. K. Malik and K. Shahzad, "MMU-OCR-21: Towards End-to-End Urdu Text Recognition Using Deep Learning," in IEEE Access, doi: 10.1109/ACCESS.2021.3110787
HandWritten_Character	821715	None	https://www.kaggle.com/datasets/vaibhao/handwritten-characters
CoronaHack -Chest X-Ray-	5933	None	https://www.kaggle.com/datasets/praveengovi/coronahack-chest-xraydataset
PANDAS Augmented Images	12083	None	https://www.kaggle.com/datasets/amyjang/pandatilesagg?select=all_images
Bacterial_Colony	681	None	https://www.kaggle.com/datasets/nilay1987/bacterial-colony
Ceylon Epigraphy Periods	5149	Colour inversion	https://www.kaggle.com/datasets/pabasar/ceylon-epigraphy-periods
Chinese Calligraphy Styles by Calligraphers	105129	None	https://www.kaggle.com/datasets/yuanhaowang486/chinese-calligraphy-styles-by-calligraphers
Graphs Dataset	15861	None	https://www.kaggle.com/datasets/sunedition/graphs-dataset

Function_Graphs Polynomial	10250	None	https://www.kaggle.com/datasets/kopfgeldjaeger/function-graphs-polynomial
sketches	20000	None	https://www.kaggle.com/datasets/vishnunkumar/sketches
Person Face Sketches	16909	None	https://www.kaggle.com/datasets/almightyj/person-face-sketches
Art Pictograms	3545	None	https://www.kaggle.com/datasets/olgabelitskaya/art-pictogram
Russian handwritten letters	219183	Monochrome and 50% colour conversion	https://www.kaggle.com/datasets/olgabelitskaya/handwritten-russian-letters https://www.kaggle.com/datasets/tatxianasnwrt/russian-handwritten-letters
Covid-19 Misinformation Tweets Labelled	13304	Monochrome, remap and 50% colour conversion	https://www.kaggle.com/datasets/arashnic/misinfo-graph
grapheme-imgs-224x224	200840	Colour inversion	https://www.kaggle.com/datasets/roycezjq/graphemeimgs224x224

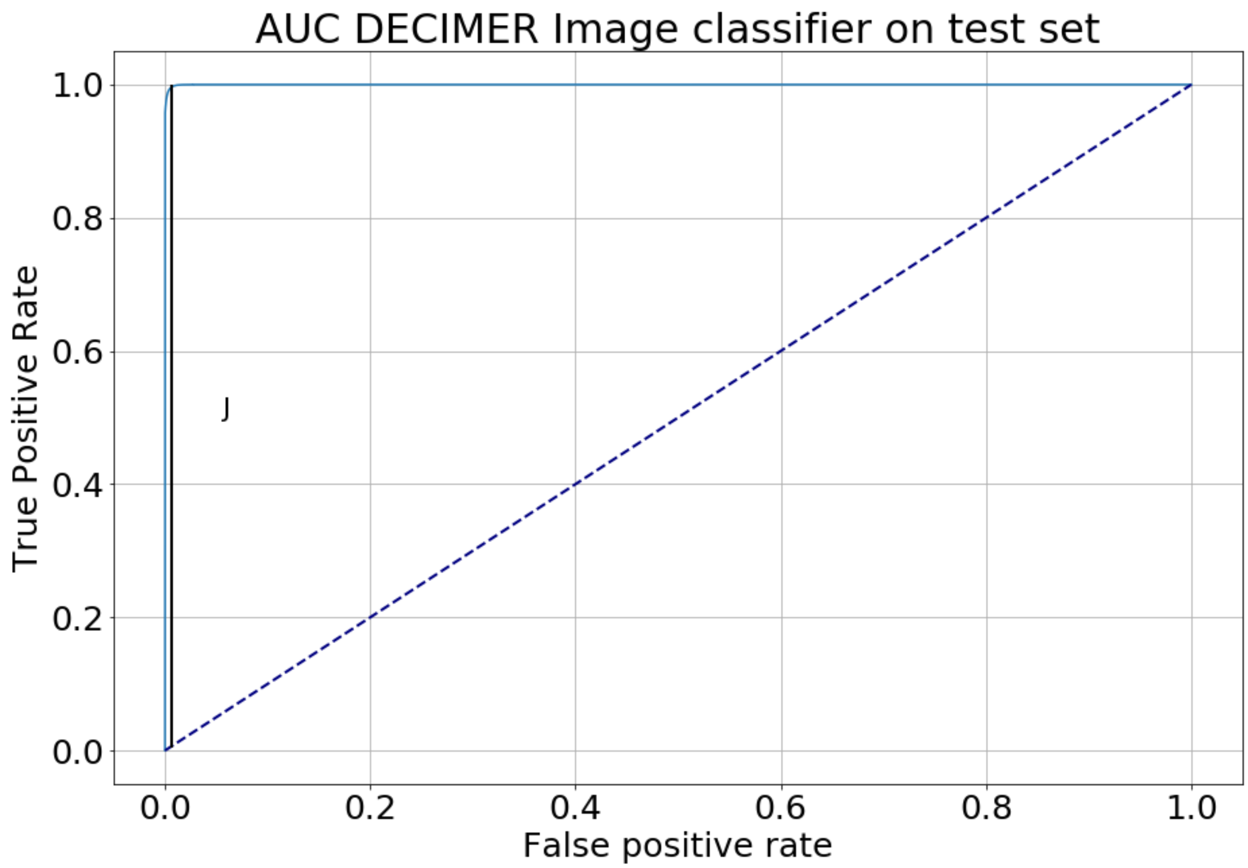

Supplementary Figure 2. AUC of the DECIMER Image classifier predictions on the test set. Dotted dark blue marks the random chance, solid light blue represents the ROC curve and the black vertical line represents the Youden index.

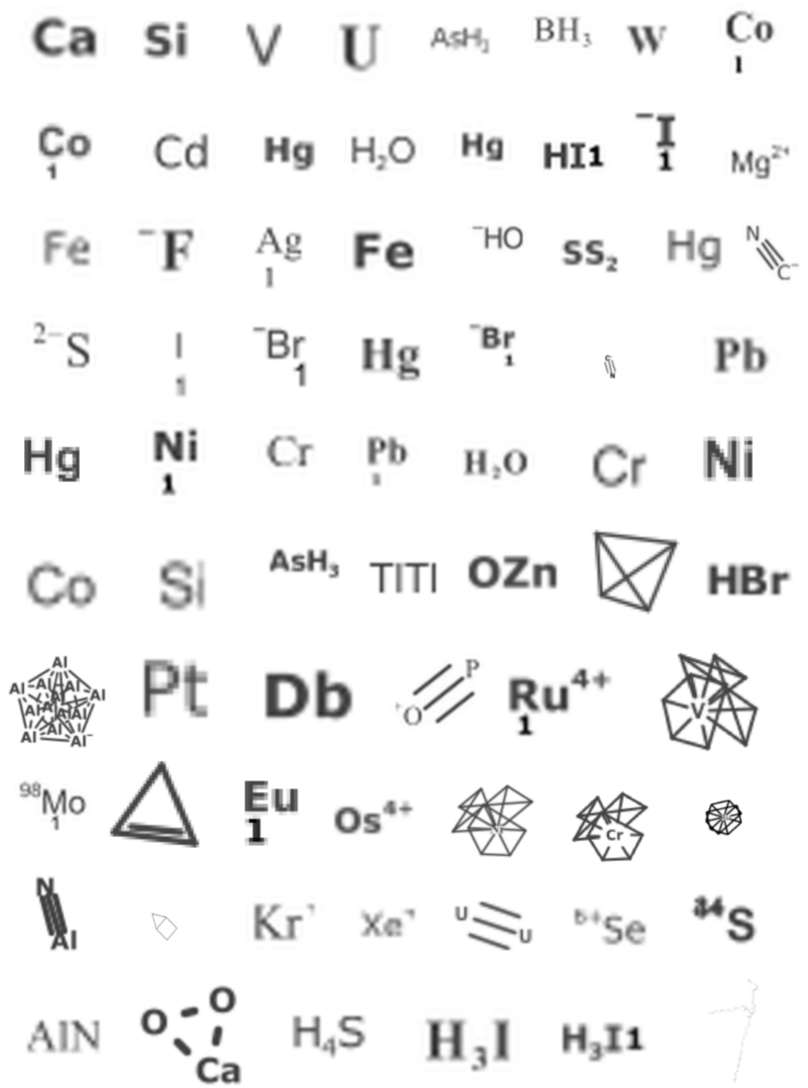

Supplementary Figure 3. Examples of non-chemical structures predicted for the ChEBI dataset.

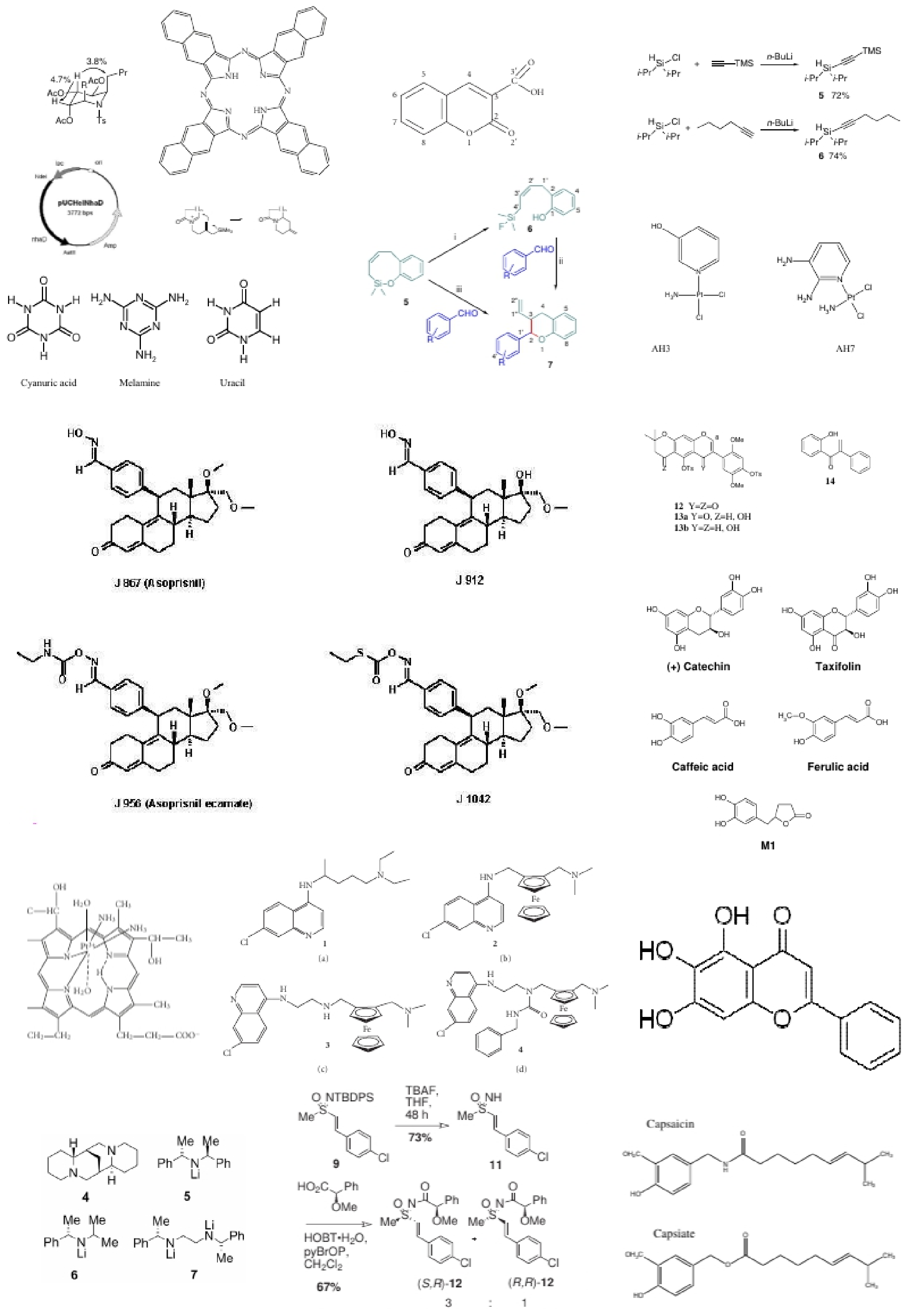

Supplementary Figure 4. Examples of chemical structures predicted on the test dataset PubLayNet.

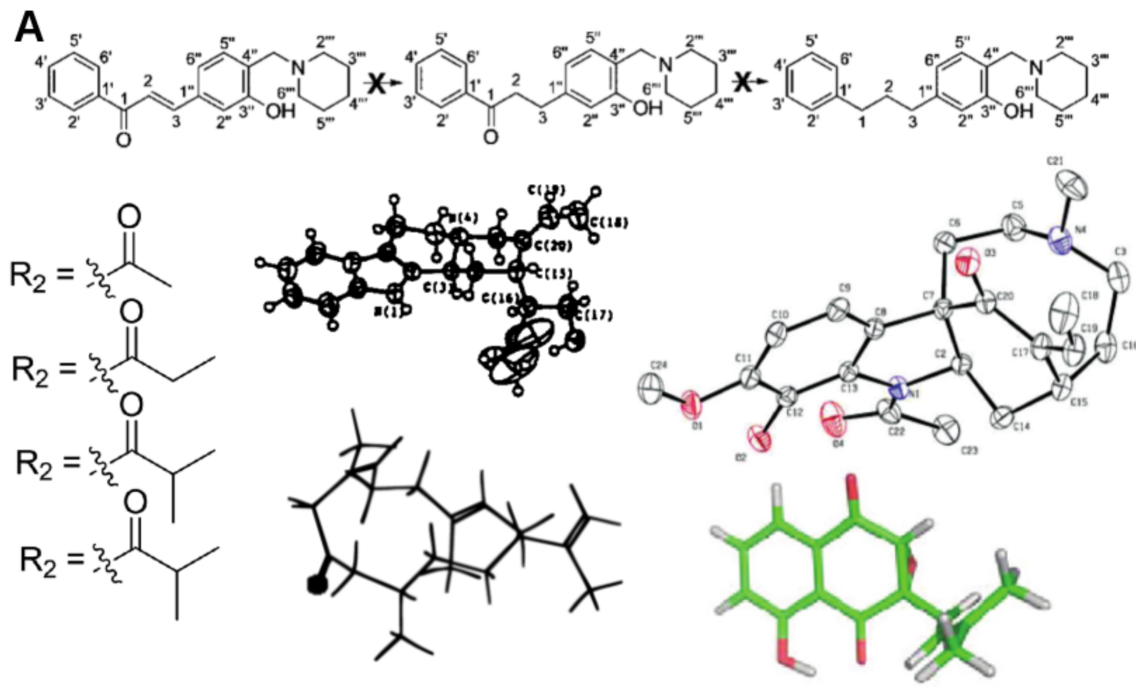

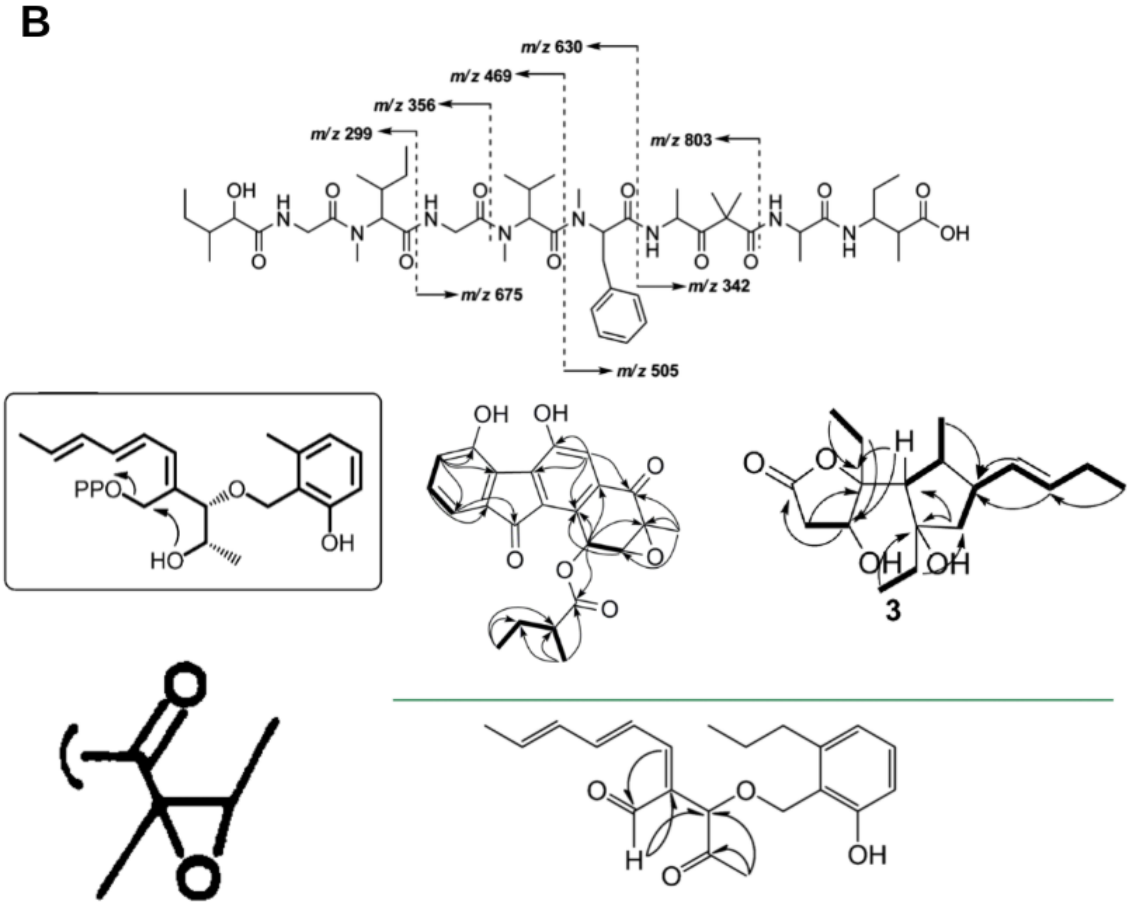

Supplementary Figure 5. Examples of wrong JNP segment classifications by the DECIMER-Image classifier. A) False positives. B) False Negative.

More detailed presentation of the DECIMER Image Classifier out-of-domain test results

Concerning the three public out-of-domain datasets, we used one that only consists of chemical structures (ChEBI), one without any chemical structures (EM_Images) and one that mostly contains no chemical structures (PubLayNet). The performance of the DECIMER Image Classifier on these datasets is as follows,

- ChEBI: 97.33% of the images were correctly classified as chemical structure depictions.
- EM_Images: 100% of the images were correctly classified as images without chemical structures.
- PubLayNet: 99.13% of the images were correctly classified as images without chemical structure depictions.

Furthermore, the performance of the DECIMER Image Classifier was evaluated using 8,733 images segmented from 1,000 publications from the Journal of Natural Products (JNP). Based on a manual inspection of the results, 8,187 of the 8,733 images were true positives, 178 were true negatives, 47 were false positives, and 321 were false negatives. As a result of computing the same performance metrics used for the test set, the results were AUC = 0.94, MCC = 0.51, accuracy = 0.96, specificity = 0.79, and sensitivity = 0.96.

It should be noted, however, that the calculated MCC was slightly lower than that calculated on the test set. It is primarily the characteristics used to determine whether an image depicts a chemical structure or not. Most of the false positive classifications are 3D chemical depictions that can not be interpreted as normal chemical structure depictions. Most of the false negative classifications are due to the presence of arrows in the image. The wrong predictions produced by the DECIMER Image Classifier are illustrated in Supplementary Figure 3-5.

Code Resource 1: Content of the script img2mol_batch_run.py that was used to run the standalone version [72] of Img2Mol in our benchmark.

```
import sys
import os
from img2mol.inference import *


def main():
    """
    This script takes three arguments:
    1) path of the directory with images to process with img2mol
    2) output directory
    3) file ending of images in the directory given at 1

    It runs img2mol on all images with the given file ending in the
    input directory and saves a file with the results in the output
    directory.
    """
    input_dir, output_dir, file_ending = sys.argv[1:]
    im_names = [img for img in os.listdir(input_dir)
                if img[-len(file_ending):].lower() == file_ending.lower()]
    img2mol = Img2MolInference(local_cddd=True)
    output_file_path = os.path.join(output_dir, "img2mol_results.smiles")
    with open(output_file_path, "w") as output_file:
        for im_name in im_names:
            im_path = os.path.join(input_dir, im_name)
            result = img2mol(filepath=im_path)
            smiles = result['smiles']
            output_file.write(f"{im_name}\t{smiles}\n")


if __name__ == "__main__":
    if len(sys.argv) == 4:
        main()
    else:
        print(f"Usage: {sys.argv[0]} input_dir output_dir file_ending")
```
